# Turning Waste into Treasure: The Full Technological Process and Product Performance Characterization of Flushable Wet Wipes Prepared from Corn Stalk

**DOI:** 10.3390/ma16227189

**Published:** 2023-11-16

**Authors:** Lulu Liu, Yeying Wang, Ziying He, Yang Cai, Kai Meng, Ke-Qin Zhang, Huijing Zhao

**Affiliations:** National Engineering Laboratory for Modern Silk, College of Textile and Clothing Engineering, Soochow University, No. 199 Ren’ai Road, Industrial Park, Suzhou 215123, China; liululu0819@163.com (L.L.); wangyeying0329@163.com (Y.W.); 20234215054@stu.suda.edu.cn (Z.H.); 2115408010@stu.suda.edu.cn (Y.C.); mk2009@suda.edu.cn (K.M.); kqzhang@suda.edu.cn (K.-Q.Z.)

**Keywords:** corn stalk, pulp board, wet-laid web formation, nonwoven materials, flushable wipes

## Abstract

As a daily consumable, wet wipes are mostly synthetic fibers, which are incinerated or landfilled after use. The nanoplastics generated during this process will lead to environmental pollution. The application of flushable wet wipes, which are dispersible and fully degradable, is of great significance. The main raw material for flushable wipes is wood pulp, which has a long growth cycle and high cost. Corn is widely planted and has a short growth cycle. Currently most corn stalk is treated by incineration, which produces a lot of smoke that pollutes the environment. Therefore, using corn stalk as the raw material for flushable wet wipes, replacing wood pulp, is both cost-effective and environmentally friendly. In this study, aiming at industrial production, we explored the full process of producing flushable wet wipes from corn stalk to pulp board, then to the final wipes. The corn stalk was treated using alkali and a bleaching agent to obtain corn stalk pulp, which was then made into pulp board through the nonwoven wet-laid process. The optimal parameters for the alkali treatment and bleaching were obtained. The properties of the corn stalk pulp board were compared with the commercial wood pulp board. Further, we mixed the corn stalk pulp with Lyocell fiber to prepare wet-laid webs, which were then bonded using a chemical binder poloxamer. Then, the evenness of the web, mechanical properties, absorption, and dispersibility of the flushable wipes were characterized. Results showed that the pulp obtained using the optimal treatment process has a high yield and better whiteness. The properties of the corn stalk pulp board are comparable with the commercial wood pulp board, which can therefore potentially be replaced by the corn stalk board prepared in our study. The prepared flushable wet wipes had good evenness and their water absorption rate was more than 600%. The mechanical strength in dry and wet states achieved 595.94 N/m and 179.00 N/m, respectively. Most importantly, the wet wipes can completely disperse under the standardized testing method. A good balance of dispersibility and wet strength of the wet wipes was achieved.

## 1. Introduction

The demand for baby wipes and toilet wipes has increased as a result of widespread concerns about hygienic conditions and sanitation. Additionally, medical disinfectant wipes are frequently used to disinfect the surfaces of commodities, furniture, etc., in big public spaces like hospitals, hotels, schools, shopping malls, etc. [1]. The increase in the use of wet wipes has also brought many problems, the major one of which is the disposal problem after their use. Wet wipes are primarily made of synthetic fibers, which can exist in the environment for up to 100 years. Some people discard wet wipes in the environment after use, or due to improper supervision and handling, the wipes enter atmospheric circulation, and after continuous environmental weathering, they are broken down into tiny structures to form microplastics [2] and then form nanoplastics [3]. Cell membranes can be penetrated by these nanoplastics, causing damage to human liver and lung cells. At the same time, the heavy use of wipes increases the amount of waste in landfills and incinerators, while landfill leachate and incineration residues also release large amounts of microplastics into the environment [4]. Microplastics can be ingested by organisms and thus enter the food system, posing a serious threat to human health [5,6].

Flushable wipes that can be flushed out, dispersed, and biodegraded can address the issue of treating wet wipes after use. The original flushable wipes, now called dimensional flushable wipes, were achieved by reducing the wipes’ size; they can only be flushed and are not fully dispersible and degradable. The problem of degradability can be tackled by the raw material selection. Natural cellulose fibers and other degradable raw materials can achieve the complete degradation of wet wipes. The dispersibility of wet wipes is connected to the length of the fiber in the raw materials, the reinforcement method, the reinforcement conditions, etc. In general, wet wipes with a low wet strength have good dispersibility, but the wet strength cannot be too low and must fulfill the basic requirements for use. Therefore, achieving a balance between wet strength and dispersibility is a difficult and critical issue for flushable wipes. Li [7], Gao [8], and co-workers used wood pulp/viscose as the raw material and spunlacing technology, improving the strength of the web by entangling fibers with each other. However, spunlacing requires a certain fiber length. Zhang and co-workers [9] studied the influence of water jet pressure on flushable wipes with different fibers and spunlacing processes and found that complete dispersion could not be achieved for some products. Deng and co-workers [10] developed a new spunlacing technology to consolidate the fiber web, which can be easily dispersed when compared with conventional carded hydroentangled nonwovens. The wet tensile strength and tensile properties of the samples were also improved. Zhang and co-workers [11] studied the wet strength, softness, and dispersibility of wet wipes prepared using wood pulp and Danufil fiber. The results showed that with the increase in Danufil fiber content or their length-to-diameter ratio, the wet strength of the wipes increased and the dispersibility decreased. The dispersibility is inversely proportional to the wet strength, according to the research reported by Atasagun and co-workers [12], who also demonstrated that the production technology has a major impact on the structure, mechanical properties, and flushability of nonwovens. According to the studies of Durukan [13] and co-workers, the flushability of wet wipes can be achieved by using plant fibers and certain consolidating structures. The addition of ES fiber and thermal bonding can lead to insufficient dispersibility of wet wipes [14]. In addition, dispersibility-aging of flushable wet wipes happened during storage, the reasons for which included swelling, ionic shielding, and inter-diffusion [15]. Chemical bonding uses adhesives to stabilize the web structure and meet the requirements of use. Some scholars use temperature-sensitive adhesives to achieve a balance between the wet strength and the dispersibility of the flushable wipes through the phase transformation of the adhesive at different temperatures. Inspired by mussels, Xu [16] and co-workers studied citrate tissue adhesives, which have a significant temperature sensitivity. Kim [17] and co-workers investigated a temperature-sensitive anti-sticking poloxamer hydrogel that can reduce adhesions after total knee arthroplasty. Although the above techniques and methods have not been widely used in the field of flushable wipes, these studies provide some theoretical basis for our research.

According to previous research, the use of plant fiber as the main raw material for wipes can achieve the ideal dispersibility. Among them, the research on wood pulp fiber is the most numerous and mature; however, the preparation cycle of wood pulp fiber is long, the shortest time is 3–5 years, and it needs to be cultivated by specialists. Therefore, other cellulose fibers are considered as alternatives to wood pulp fibers, which can also achieve the complete degradability of flushable wipes. Corn is planted in a vast area, the largest area of all the food crops, up to 107 million acres in China. Furthermore, corn has a much shorter growth cycle than trees—the shortest is only 75 days—but its cellulose and hemicellulose content can be comparable to wood [18]. Due to its short growth cycle, farmers plant a large amount of corn, which results in a lot of waste corn stalks that cannot be disposed of and must instead be burnt. This not only creates a large amount of waste but also a lot of smoke and toxic gases that are harmful to the environment and the human body.

In this study, we propose to replace wood pulp fiber with corn stalk fiber in the preparation of flushable wipes, which will not only provide a rich source of raw materials for flushable wipe research but will also tackle the environmental pollution problem caused by stalk burning. Furthermore, corn stalk fiber has a relatively larger length-to-diameter ratio and higher strength, which has certain advantages for the nonwoven wet-laid web formation process [19], promoting the subsequent preparation of the pulp board and wet wipes.

## 2. Materials and Methods

### 2.1. Materials and Reagents

The corn stalk used in this study was produced in Shijiazhuang City, Hebei Province, China. Sodium hydroxide (≥98%, NaOH) and sodium hypochlorite solution (4%, 5%, 6%, NaClO) were purchased from UTOP Technology Suzhou Co., Ltd., Suzhou, China. Hydrogen peroxide solution (5%, H_2_O_2_) was purchased from Blue Lake Chemical Reagent Co., Ltd. (Hangzhou, China) Sulphuric acid (analytically pure, H_2_SO_4_) was received from Yonghua Chemical Technology (Jiangsu) Co., Ltd., Changshu, China. Degreased gauze was obtained from Jiangsu Argon Krypton Xenon Material Technology Co., Ltd., Suzhou, China. Lyocell fibers (1.25 dtex × 6 mm) were obtained from Hangzhou Youbiao Technology Co., Ltd., Hangzhou, China. Poroxamer (F127 NF) was purchased from Shandong Yousuo Chemical Technology Co., Ltd., Heze, China.

### 2.2. Methods

#### 2.2.1. Preparation of Corn Stalk Pulp

To obtain corn stalk pulp, corn stalk needs to be prepared by pre-treatment, alkali treatment, bleaching and cleaning. Corn stalk was cut into 1 cm stalk segments with a chopper knife. The corn stalk (5 g) was soaked in deionized water (150 mL), heated in a water bath at 100 °C for 20 min, and then heated at 80 °C for 100 min. The alkali treatment was carried out to investigate the effect of alkali concentration, time, and temperature on the corn stalk pulp. There were 3 parallels in each set of experiments. In the bleaching experiment, the effect of sodium hypochlorite (NaClO) concentration on corn stalk pulp was discussed, and a three-factor, three-level orthogonal experimental scheme was designed to adjust the bleaching time (A), bleaching temperature (B), and pH (C). To reduce the bleaching concentration for the sake of environmental protection and labor protection, each group of experiments was repeated 3 times. Finally, 5% hydrogen peroxide solution (H_2_O_2_) was used for secondary bleaching for 1 h and washed with deionized water.

#### 2.2.2. Preparation of Corn Stalk Pulp Boards

Corn stalk pulp of 80 g was mixed with 2 L deionized water, and beat in two batches using a standard fiber dissociator (IMT-SJ02, Dongguan International Material Tester Co., Ltd., Dongguan, China) at 4000 rpm for 4 min. After beating, the slurry was poured into a sheet former (CP03A, Dongguan International Material Tester Co., Ltd.), blown, and stirred for 30 s, followed by rapid drainage and vacuum dewatering for 30 s. The web was subsequently dried in oven at 60 °C for 5 h. A 30 cm × 30 cm pulp board could be prepared from 80 g of corn stalk pulp, and a total of 3 samples were prepared for subsequent testing and characterization.

#### 2.2.3. Preparation of Flushable Wipes

Corn stalk pulp (Co) and Lyocell fiber (Ly) were mixed in the ratios of 10:0, 7:3, 5:5, 3:7, and 0:10 (each ratio has 3 parallels), respectively. Co/Ly blend (5 g) and deionized water (300 mL) was mixed and then beaten with a high-speed homogenizer (FJ200-S, Shanghai Lichen Bangxi Instrument Technology Co., Ltd., Shanghai, China) at 5000 r/min. The pulping was considered complete when no fiber tangles or knots were observed. After pulping was completed, a 400-mesh screen was used for wet laying. Fibers were evenly dispersed in the water during web formation. Then, the dehydrated web was dried in oven at 60 °C until its mass was constant.

In order to study the influence of poloxamer content on the wet strength and dispersibility of the flushable wipes, samples with different poloxamer concentrations were prepared. The webs were prepared with a ratio of 7:3 of corn stalk and Lyocell fiber; then, the webs were immersed in poloxamer adhesive with concentrations of 7.5%, 10%, and 12.5%, respectively, while shaking to make the adhesives disperse evenly. After the binder was completely absorbed by the webs, the samples were dried to form flushable wipes.

### 2.3. Characterization

#### 2.3.1. Characterization of Corn Stalk Pulp

##### Yield of Corn Stalk Pulp

After pre-treatment, alkali treatment, and bleaching of corn stalk, the corn stalk pulp was washed with deionized water to reduce the pH to 7, then dried and weighed. The yield value of corn stalk pulp was calculated according to Equation (1). Three parallel tests were carried out to obtain the average result.

(1) $$R=\frac{m_{1}}{m_{2}}\times 100$$

where R was the corn stalk pulp yield (%), m_1_ was the mass of corn stalk after drying (g), and m_2_ was the mass of corn stalk before treatment (g).

##### Whiteness

We used a colorimeter (ULtraScan PRO, HunterLab, Reston, VA, USA, the accuracy is 0.01) to test the relative whiteness of the prepared corn stalk pulp. The conditions of the instrument were checked and calibrated before testing. Six different parts of the sample were selected to test their whiteness, and the data were averaged to obtain the final whiteness value.

#### 2.3.2. Characterization of Corn Stalk Pulp Board

##### Morphology

Microstructure, fiber morphology, and changes in surface morphology of corn stalk pulp boards were observed using scanning electron microscopy (Regulus 8100, Hitachi Division, Tokyo, Japan) operated at 3 kV. The samples were coated with gold for 90 s prior to the SEM studies to improve their electrical conductivity.

##### Fourier Transform Infrared Spectroscopy (FTIR)

Two milligrams of untreated, pre-treated, alkali-treated, and bleached corn stalk powder were weighed and ground with 100 mg of potassium bromide (KBr) crystals, respectively, and pressed into circular sheets of about 1 mm thickness. FTIR spectrometer (Nicolet 5700 FTIR, Nicolet, Green Bay, WI, USA) was employed for recording the FTIR spectra of different treatment stages.

##### X-ray Diffractometer (XRD)

Changes in the crystalline structure of corn stalk before and after treatment were analyzed using an X-ray diffractometer (X’Pert PRO MPD, Malvern Panaco, Amsterdam, The Netherlands) operated at a scanning angle of 3°−60°and a scanning speed of 1°/min.

##### Mechanical Properties

The specimens were cut into 250 mm × 50 mm size and the tensile strength of the corn stalk pulp boards was measured using universal testing machine (Instron 5967, Instron Corporation, Norwood, MA, USA, load and displacement values are accurate to 0.00001) operated at a gauge length of 200 mm and a crosshead speed of 100 mm/min. The value in tensile strength and in elongation at break were calculated according to Equations (2) and (3).

(2) $$\sigma =\frac{F}{b\times d}$$

where σ was the breaking strength (MPa), F was the maximum amount of force needed to break a specimen (N), b was the width of the specimen (mm), and d was the thickness of the specimen (mm).

(3) $$\varepsilon =\frac{L-L_{0}}{L_{0}}\times 100$$

where ε was the elongation at break (%), L was the length of the specimen at break (mm), and the L_0_ was the original length of the specimen (mm).

##### Component Analysis

The content of moisture, alpha-cellulose, and ash were analyzed. The sample was dried in oven at 105 ℃ until its mass was constant. Moisture content can be calculated according to the weight loss. For measuring the alpha-cellulose content, the dried corn stalk pulp was partly dissolved in sodium hydroxide (NaOH) aqueous solution with a concentration of 17.5 wt%. Then, the solution was placed in water bath to mercerize at 20 °C for 45 min. After adding 30 mL deionized water, the slurry was poured into a crucible with fritted disc, washed by sodium hydroxide (NaOH) aqueous solution with a concentration of 9.5 wt% and deionized water until its pH was 7, and then filtered by a vacuum pump. Then, the remaining slurry was dried in oven at 105 °C until the mass was constant. The alpha-cellulose content of the corn stalk pulp was calculated using Equation (4).

(4) $$X=\frac{100\times (m_{1}-m)}{m_{2}\times (100-W)}\times 100$$

where X was the alpha-cellulose content of the corn stalk pulp (%), m_1_ was the mass of crucible and alpha-cellulose (g), m was the mass of the crucible (g), m_2_ was the mass of the sample (g), and W was the moisture content of the sample (%). For measuring the ash content, the dried corn stalk pulp was put in a muffle furnace at 575 °C for at least 4 h, and the residue was weighed.

#### 2.3.3. Characterization of Flushable Wipes

##### Thickness and Mass per Unit Area

The thickness test method refers to GB/T 24218.2-2009 “Textile–Test method for nonwovens–Part 2: Determination of thickness” [20]. For thickness measurement, the sample was placed on the reference plate and a pressure of 100 cN was applied for 10 s using a circular presser foot parallel to the reference plate. The distance between the two plates was the thickness of the sample. Each sample was measured 10 times to obtain an average result and the coefficient of variation of the thickness was calculated according to Equation (5).

(5) $$C\cdot V=\frac{\sigma }{\bar{x}}$$

where C·V was the coefficient of variation, σ was the standard deviation (mm), and $\bar{x}$ was the mean value of thickness (mm).

The mass per unit wipes test method refers to GB/T 24218.1-2009 “Textile Nonwovens Test Methods Part 1: Determination of Mass per Unit Area” [21]. To measure the mass per unit area, all the samples were cut into 50 mm × 50 mm and weighed. The mass per unit area of the sample was calculated according to Equation (6). Six samples were measured to obtain average data. The coefficient of variation of the basis weight was calculated according to Equation (5).

(6) $$G=\frac{m}{50\times 50}\times 10^{6}$$

where G was the mass per unit area of web (g/m^2^), and m was the mass of web after drying (g).

##### Optical Image Analysis of the Web

To further characterize the evenness of the nonwoven web, the sample was photographed on a black background plate under the stationary illuminant at the same location and height. ImageJ software (https://ij.imjoy.io/ (accessed on 8 November 2023)) was used to process and analyze the images, and the grayscale histograms were obtained due to the brightness and darkness of the light passing through the web at different locations.

##### Mechanical Properties

The samples were cut into 150 cm × 25 cm strips and the dry breaking force was measured using universal testing machine (Instron 5967, Instron Corporation, Norwood, MA, USA, load and displacement values are accurate to 0.00001) operated at a gauge length of 100 mm and a stretching speed of 50 mm/min. The tensile strength in the dry state was calculated according to Equation (7). The tensile strength in the wet state was measured after the specimen was completely and uniformly wetted, which was calculated according to Equation (7).

(7) $$S=\frac{F}{w_{i}}\times 10^{3}$$

where S was the tensile strength (N/m), F was the maximum force at break (N), and w_i_ was the width of the sample (mm).

##### Dispersibility

For the dispersibility of wet wipes, please refer to the decomposition experiment of the shaking box in GB/T 40181-2021 “Test Method and Evaluation of Dispersibility of Nonwoven Materials for Disposable Hygiene” [22]. A dispersibility tester (YS-381, Xi’an Bohui Instrumentation Co., Ltd., Xi’an, China) was used to evaluate the dispersibility of the wipes. The samples were placed in a shaking box containing 2 L of water and shaken at a speed of 26 rpm for 60 min; then, the tested material was poured into a screen with 12.5 mm aperture, the residual samples were collected and weighed after drying. Dispersion rate was calculated according to Equation (8). Three samples were measured for each group to obtain an average result.

(8) $$X=\frac{m_{0}-m_{1}}{m_{0}}\times 100$$

where X was the dispersion rate (%), *m*_0_ was the initial sample mass (g), and *m*_1_ was the mass of the residues on the screen (g).

##### Absorption

The absorption test adopts the liquid absorption method, and the test method refers to GB/T 24218.6-2010 “Test Methods for Textiles Non-Woven Fabrics Part 6: Determination of Absorption” [23]. The samples prepared in our study and obtained commercially were cut into a size of 100 mm × 100 mm, and the mass of the sample before and after liquid absorption was weighed. The liquid absorption of the sample was calculated according to Equation (9).

(9) $$Y=\frac{m_{1}-m_{0}}{m_{0}}\times 100$$

where Y was the liquid absorption rate (%), *m*_0_ was the initial mass of the sample (g), and *m*_1_ was the mass of the sample after absorbing liquid (g).

#### 2.3.4. Data Analysis

Origin 2021 software was used for graphing and the results are expressed as mean ± standard deviation (SD). Tukey’s tests were used to analyze the differences between groups. Stars represent a significant difference (*p* < 0.05), specifically, *, **, ***, and **** mean 0.01 < *p* < 0.05, *p* < 0.01, *p* < 0.001, and *p* < 0.0001, respectively, and ns means there are no significant differences.

## 3. Results and Discussion

### 3.1. Alkali Treatment and Bleaching of Corn Stalk Pulp

The pre-treated corn stalk was treated with alkali, the sodium hydroxide concentrations were 13%, 16%, 19%, 22%, and 25%, the alkali treatment times were 40 min, 50 min, and 60 min, and the alkali treatment temperatures were 70 °C, 80 °C, and 90 °C, respectively. The orthogonal experiments showed the optimal treatment conditions were an alkali concentration of 19%, an alkali treatment time of 50 min, and an alkali treatment temperature of 90 °C (see Supporting Information). Due to the fact that the whiteness of corn stalk pulp decreases with the increase in alkali concentration, the highest whiteness was obtained by bleaching with an alkali concentration of 19%. Under the optimal alkaline treatment conditions, three factors and three levels of orthogonal experiments were conducted, and the optimal bleaching treatment conditions were obtained.

The combination with the highest yield under similar whiteness conditions was treated at a temperature of 15 °C, a pH of 7, and a time of 60 min (see Supporting Information for a detailed discussion).

**Figure 1:**
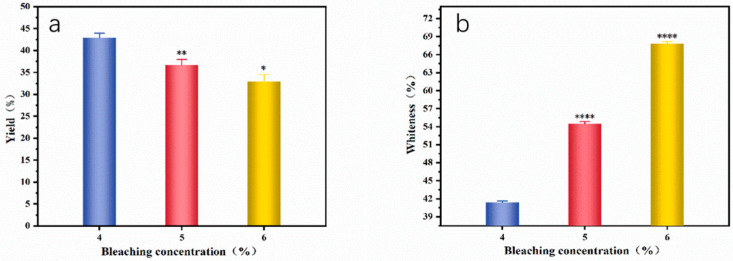
The effect of bleaching concentration on the yield (**a**) and whiteness (**b**) of corn stalk pulp. Stars represent a significant difference (*p* < 0.05), specifically, *, **, and **** mean 0.01 < *p* < 0.05, *p* < 0.01, and *p* < 0.0001, respectively.

The bleaching process uses sodium hypochlorite solution to saturate part of the double bond in the pigment to achieve a neutral color. Here, not only are the color-developing substances removed but the corn stalk will also be subjected to certain oxidation [24,25]. Therefore, the higher the concentration of the sodium hypochlorite solution, the lower the yield of the corn stalk pulp, as shown in Figure 1a.

The higher the bleaching concentration, the higher the whiteness of corn stalk pulp. When the concentration of sodium hypochlorite solution is 6%, the pulp has the best whiteness, as shown in Figure 1b. The higher the effective chlorine content during the bleaching process, the stronger the bleaching effect on the corn stalk, resulting in an increase in the whiteness of the pulp.

### 3.2. Performance of the Corn Stalk Pulp Board

#### 3.2.1. Morphology of the Corn Stalk Pulp Board

**Figure 2:**
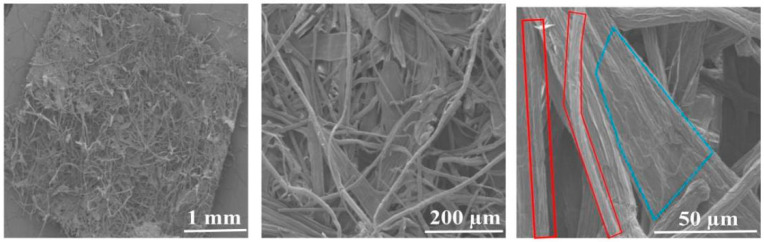
Morphology of corn stalk pulp board. The corn stalk fiber is framed by the red line, the corn stalk core is framed by the blue line.

As shown in Figure 2, it can be observed that there are two parts to the pulp board (the corn stalk fiber is framed by the red line, the corn stalk core is framed by the blue line). The corn stalk fiber is thinner, has a larger aspect ratio, and has vertical lines on the surface. Additionally, the corn stalk core, adhering to the corn stalk fibers, acts as a bonding and reinforcing material in the corn stalk pulp board, which is beneficial for improving the mechanical properties of the pulp board.

#### 3.2.2. Structure and Crystallinity

**Figure 3:**
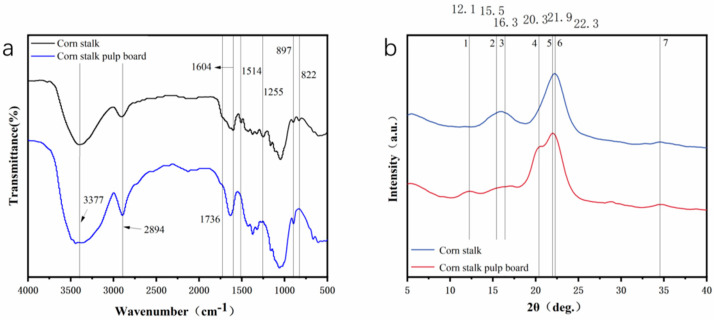
Infrared curve (**a**) and XRD spectrum (**b**) of corn stalk pulp board.

Corn stalk mainly contains cellulose, hemicellulose, lignin, etc. Figure 3a shows the Fourier transform infrared (FTIR) spectrum of the corn stalk pulp board before and after treatment. The peaks at 1604 cm^−1^, 1514 cm^−1^, and 822 cm^−1^ could be assigned to the lignin aromatic skeleton vibration and the C=O stretching vibration and these peaks disappeared in the curve of the treated corn stalk pulp board, indicating the removal of lignin. For hemicelluloses, the peak at 1736 cm^−1^ attributed to the acetyl group also disappeared after treatment, indicating the removal of hemicelluloses. The characteristic peak of the C–O–C bond at 1255 cm^−1^ disappeared, indicating that the hemicellulose and lignin had been removed. The peaks at 1640 cm^−1^, 1514 cm^−1^, and 822 cm^−1^, attributed to the O–H stretching vibration, C–H stretching vibration, and the characteristic peak of the cellulose-β-D-glucoside, show little change before and after treatment, indicating that the treatment process causes little damage to cellulose [18,26].

The different lattice planes corresponding to cellulose I and cellulose II in different 2θ are shown in Table 1. As shown in Figure 3b, the corresponding lattice plane of the corn stalk pulp board changes from cellulose I to cellulose II. Using the peak fitting method to calculate the crystallinity of corn stalk and corn stalk pulp board, the results were 47.03% and 44.06%, respectively. The crystallinity slightly decreases, and the amount of crystallinity will influence the moisture absorption and mechanical properties of the pulp board.

#### 3.2.3. Mechanical Properties

The tensile strength of the corn stalk pulp board is 2.44 MPa, and the elongation at break is 2.94%. Corn stalk fibers are finer and become more compact after being made into pulp board. Corn stalk core plays a crucial role in improving the tensile strength of the pulp board due to it attaching corn stalk fibers together as an adhesive to form a nonwoven bonded structure. Meanwhile, the vertical grooves on the fiber’s surfaces increased the friction between fibers, resulting in tighter entanglement, which is beneficial to improve the strength of the pulp board.

The Young’s modulus of the corn stalk pulp board was 6.89 MPa, calculated through linear fitting. The smaller the Young’s modulus, the softer the material is. Thus, the corn stalk pulp board has excellent bending performance which makes it easy to transform into a coiled material that is convenient to transport.

#### 3.2.4. Components of Corn Stalk

The main components of the corn stalk pulp board are shown in Table 2. The content of alpha-cellulose in corn stalks is relatively high, which is beneficial for moisture absorption. What is more, the decrease in crystallinity, the grooves on the fiber surfaces, and the porous structure of the corn stalk core also result in better moisture absorption.

### 3.3. Performance of the Flushable Wet Wipes

#### 3.3.1. Uniformity of the Flushable Wet Wipes

The uniformity of the flushable wet wipes can be reflected by the uniformity of the thickness, mass per unit area, and optical images.

The thickness of the web is between 0.25 mm and 0.35 mm, and the web thickness shows a trend of increasing first and then decreasing. Because corn stalk core has a connecting and supporting effect on the web, as the amount of Lyocell fibers increases in the web, the fibers become entangled, and the thickness of the web increases. If webs contain 100% Lyocell fibers, there is no adhesion between the fibers, resulting in a decrease in the thickness of the web.

The mass per unit area of the web is about 50 g/m^2^, and with the increase in Lyocell fiber, the mass per unit area of the web increases slightly. Considering that the short length and fineness of the corn stalk pulp can easily cause losses during the forming process, increasing the Lyocell fibers entangling with the corn stalk pulp, reduces the loss of corn stalk pulp and increases the mass per unit area of the fiber web slightly.

**Figure 4:**
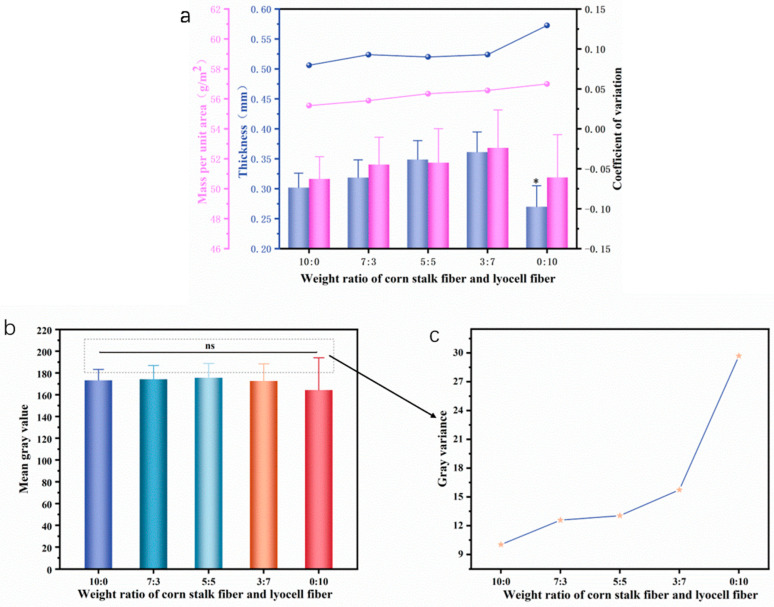
Uniformity of fiber web. (**a**) The influence of fiber ratio on web thickness and mass per unit area. (**b**) The average gray value of different fiber ratios. (**c**) The gray variance. Stars represent a significant difference (*p* < 0.05), specifically, * 0.01 < *p* < 0.05, and ns means there are no significant differences.

As shown in Figure 4, the thickness and mass per unit area variation coefficient of the web increased with an increase in Lyocell fibers. The larger the coefficient of variation, the greater the difference in various parts of the web, reflecting a decrease in the uniformity of the web. This is because Lyocell fibers have a longer length and are entangled with the corn stalk pulp refining process, which is not beneficial to the dispersion of the pulp and leads to a decrease in the uniformity of the fiber web.

Through optical images, grayscale histograms can be obtained. The mean gray value reflects the brightness of the image, while the gray variance reflects the contrast of the image. The larger the variance, the greater the difference in grayscale between each pixel. As shown in Figure 4b,c, the difference in the mean gray value of the fiber web is not significant, approximately at around 170. However, with the increase in Lyocell fibers, the variance of grayscale significantly increases, indicating that with the increase in Lyocell fibers, the uniformity of the wipe becomes worse. Shorter fiber length is beneficial to web uniformity because they can disperse evenly during the wet-laid process.

#### 3.3.2. Mechanical Properties

**Figure 5:**
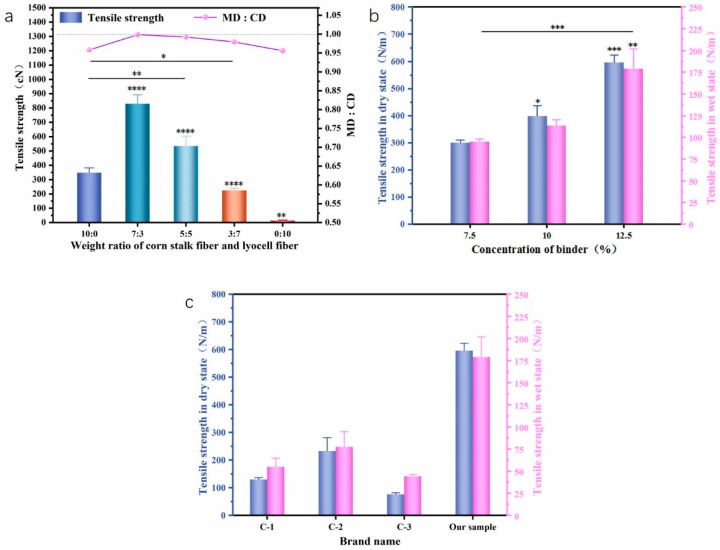
Mechanical properties of wet wipes. (**a**) The breaking strength and longitudinal and transverse strength ratio of the fiber web with different fiber ratios. (**b**) The effect of different adhesive concentrations on the mechanical properties of wet wipes. (**c**) The strength comparison of commercially available flushable wet wipes with our samples. Stars represent a significant difference (*p* < 0.05), specifically, *, **, ***, and **** mean 0.01 < *p* < 0.05, *p* < 0.01, *p* < 0.001, and *p* < 0.0001, respectively.

As shown in Figure 5a, with the content increase in Lyocell fiber, the tensile strength of the web increased first and then showed a decreasing trend. The wipe with a corn stalk and Lyocell fiber ratio of seven to three has the maximum tensile strength. That is because more corn stalk pulp contains more corn stalk core, which provides more adhesive points, leading to a higher tensile strength. This can explain the reason that wipes with pure Lyocell fibers have the lowest tensile strength. However, fiber length is also important for improving the tensile strength of the wipes because longer fibers are easier to entangle between themselves. The increase in tensile strength depends on the synergistic effect of the adhesive and the fiber length.

The ratio of tensile strength at machine direction (MD) and cross direction (CD) is normally used in nonwoven science to characterize web uniformity. The closer the ratio is to 1, the more isotropic the mechanical strength of the web. Figure 5a showed that the MD to CD ratio was closest to 1 when the ratio of corn stalk and Lyocell fiber was seven to three. According to the results obtained from mass per unit area, thickness, optical image, and tensile strength ratio at machine direction (MD) and cross direction (CD), it can be concluded that the wipes had optimal uniformity when the ratio of corn stalk and Lyocell fiber was seven to three.

As shown in Figure 5c, the mechanical strength of the washable wipes is significantly better than that of commercially available products when the adhesive concentration is 12.5%. By comparing the test results of the dry and wet strength of flushable wipes in other literature, our mechanical properties are also at a medium level, which meet the daily use requirements [13,27].

Wet wipes with different amounts of adhesive were also prepared to study their mechanical properties. The solubility of the poloxamer changes sharply with an increase in the environmental temperature, and this phase separation is usually considered to be caused by the equilibrium of the hydrophilic and hydrophobic parts on the polymer chain and the mixing free energy [28]. From Figure 5b, it can be seen that with an increase in the adhesive concentration, the dry and wet strength of wet wipes showed an increasing trend. This is due to the fact that the poloxamer is a PEO–PPO–PEO triblock copolymer [29]. As the temperature increases, the hydrogen bonding force between the poloxamer and the water weakens [30,31], and the epoxy propane block rapidly dehydrates into a more hydrophobic structure. The phase of the poloxamer undergoes a sudden transition, forming micelles at the critical micellization temperature [32], thereby strengthening the wet wipes and improving their mechanical properties. At the same time, the concentration of the adhesive is positively correlated with the viscosity of the solution [33]. As the concentration of the adhesive increases, the viscosity of the solution increases, and the bonding between the solution and the fibers becomes closer, resulting in an increase in the mechanical properties of the wet wipes.

#### 3.3.3. Dispersibility

**Figure 6:**
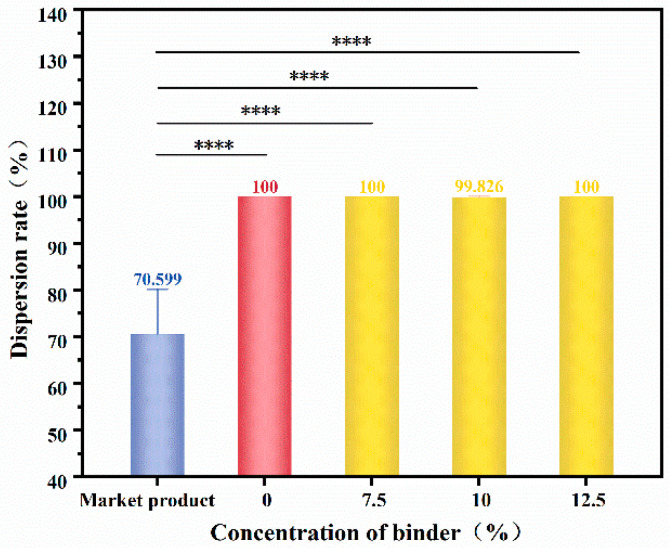
Effect of different binder concentrations on the dispersibility of wet wipes. Stars “****” mean *p* < 0.0001.

As shown in Figure 6, the dispersion rate of a flushable wet wipe is only 70.599%. For the wipes prepared in this study, a nonwoven web without a binder was used as a control, which has weak adhesion in a wet state and is easily dispersed in water. No matter how much the binder content was, the dispersion rate of the wet wipes was about 100%, indicating that they could be completely dispersed in a flushed toilet. The poloxamer has a reversible thermosensitive gelling property. When the system rises to a certain temperature, it becomes a semi-solid gel, and when the temperature decreases, it turns into a flowing liquid again [34]. Thus, the excellent dispersibility of the wet wipes prepared in our study is due to a lower temperature in the shaking box because the binder phase changes from a gel to a solution, resulting in the weakening of the adhesion force between fibers.

#### 3.3.4. Absorption Performance

**Figure 7:**
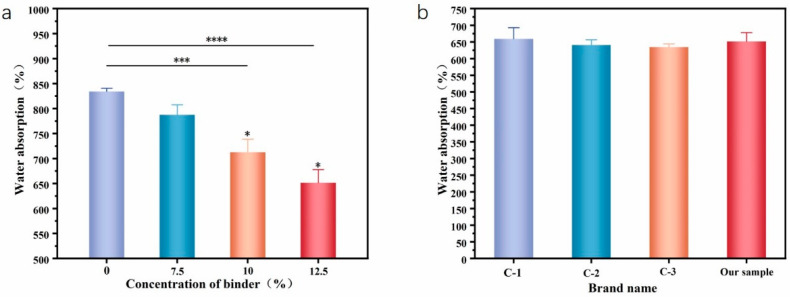
Comparison of the absorption performance of flushable wipes. (**a**) The effect of different adhesive concentrations on the absorption performance of wet wipes. (**b**) The absorption performance of different brands of flushable wipes compared to our sample. Stars represent a significant difference (*p* < 0.05), specifically, *, ***, and **** mean 0.01 < *p* < 0.05, *p* < 0.001, and *p* < 0.0001, respectively.

As shown in Figure 7a, with the increase in binder concentration, the water absorption performance of the web shows a gradually decreasing trend. Due to the poloxamer containing hydrophilic ethylene oxide units and hydrophobic propylene oxide units, it is essentially amphiphilic. The phase transition of the poloxamer is mainly due to the micelles’ formation of dehydrated propylene oxide. With an increase in the poloxamer, more and more water was bound, which led to the decreasing chemical potential of free water and the enhancement of propylene oxide dehydration, thereby the formation of micelles was accelerated and the critical micellization temperature was decreased [35]. Therefore, with an increasing temperature and concentrations of poloxamer, the hydrophobic chain conformation is easier to form, and the hygroscopicity of the wipes decreases. The water absorption rate of the wipes without a binder is 834.23%, and the water absorption rate of the wet wipe with a 12.5% binder concentration is 625.24%. Figure 7b shows the comparison of the absorption performance of the flushable wipes with a 12.5% adhesive and commercially available flushable wipes (brand names are anonymous and presented by C-1, C-2, C-3). According to the data, our absorption performance is close to that of the commercially available products.

## 4. Conclusions

In this study, corn stalks were used as a raw material to explore the whole process of making wipes: from pulp to pulp board to washable wipes. The optimal preparation process of the corn stalk pulp was obtained, including the alkali treatment and the bleaching parameters. The performance of the corn stalk pulp board is comparable to that of a commercialized wood pulp board. With 12.5% poloxamer as a binder, the wet wipes prepared (in a ratio of corn stalk fiber to Lyocell fiber of seven to three) can be completely dispersed. More importantly, their tensile strength in a wet state reached 179.00 N/m, which meets the requirements for actual use. The liquid absorption of the wipes was 625.24%, which was comparable to the commercially available products. In summary, the flushable wet wipes prepared in this study take advantage of the characteristics of temperature-sensitive adhesives to maintain their wet strength while ensuring 100% dispersibility, achieving a balance between flushable performance and wet strength, which realizes the transformation of corn stalk from an agricultural waste resource into a treasure. Alkali is used to treat the raw corn stalk. Although the amount of alkali was controlled to be as little as possible in the current process, we still think that it is better to reduce the number of chemicals in further studies for the sake of environmental protection and cost reduction.

## Figures and Tables

**Table 1:** Cellulose I and cellulose II are different 2θ corresponding crystal planes.

Number	Cellulose Ⅰ	Number	Cellulose Ⅱ
2	110 (15.5)	1	110 (12.1)
3	110 (16.3)	4	110 (20.3)
6	200 (22.3)	5	200 (21.9)
7	004 (34.5)		

**Table 2:** Analysis of main components of corn stalk pulp board.

	Alpha-Cellulose (%)	Moisture Content (%)	Ash Content (%)
Corn stalk pulp board	89.95	4.63	0.71

## Data Availability

Data can be found from corresponding author.

## Supplementary Materials

The following supporting information can be downloaded at: https://www.mdpi.com/article/10.3390/ma16227189/s1. Figure S1. Effect of alkali treatment process on yield and whiteness of corn stalk pulp. Refs. [36,37] are cited in the Supplementary Materials.

## References

[1] Shruti V.C., Pérez-Guevara F., Kutralam-Muniasamy G. (2021). Wet wipes contribution to microfiber contamination under COVID-19 era: An important but overlooked problem. Environ. Chall..

[2] Munoz L.P., Baez A.G., McKinney D., Garelick H. (2018). Characterisation of “flushable” and “non-flushable” commercial wet wipes using microRaman, FTIR spectroscopy and fluorescence microscopy: To flush or not to flush. Environ. Sci. Pollut. Res..

[3] Lee J., Jeong S., Chae K.J. (2021). Discharge of microplastics fibres from wet wipes in aquatic and solid environments under different release conditions. Sci. Total Environ..

[4] Silva A.L.P., Prata J.C., Duarte A.C., Soares A.M.V.M., Rocha-Santos T. (2021). Microplastics in landfill leachates: The need for reconnaissance studies and remediation technologies. Case Stud. Chem. Environ. Eng..

[5] Lima A.R.A., Ferreira G.V.B., Barrows A.P.W., Christiansen K.S., Treinish G., Toshack M.C. (2021). Global patterns for the spatial distribution of floating microfibers: Arctic Ocean as a potential accumulation zone. J. Hazard. Mater..

[6] Kutralam-Muniasamy G., Pérez-Guevara F., Elizalde-Martinez I., Shruti V.C. (2020). An overview of recent advances in micro/nano beads and microfibers research: Critical assessment and promoting the less known. Sci. Total Environ..

[7] Li W.A., Liu N., Nie D., Li S.Y. (2020). Study on performance and material of flushable wet wipes. Text. Rep..

[8] Gao J.Y., Wu H.B. (2012). Manufacture Technology of Flushable Material Used for Wet Wipes. China Pulp Pap..

[9] Zhang Y.J., Xu Y.K., Zhao Y., Huang C., Jin X.Y. (2018). Effects of short-cut fiber type and water-jet pressure sum on wet strength and dispersibility of wood pulp-based wetlaid/spunlace wipes. Eur. J. Wood Wood Prod..

[10] Deng C., Liu W.J., Zhang Y.J., Huang C., Zhao Y., Jin X.Y. (2018). Environmentally friendly and breathable wet-laid hydroentangled nonwovens for personal hygiene care with excellent water absorbency and flushability. R. Soc. Open Sci..

[11] Zhang Y.J., Deng C., Wang Y.X., Huang C., Zhao Y., Jin X.Y. (2019). A new dispersible moist wipe from wetlaid/spunlace nonwoven: Development and characterization. J. Ind. Text..

[12] Atasagun H.G., Bhat G.S. (2020). Advancement in flushable wipes: Modern technologies and characterization. J. Ind. Text..

[13] Durukan S., Karadagli F. (2019). Physical characteristics, fiber compositions, and tensile properties of nonwoven wipes and toilet papers in relevance to what is flushable. Sci. Total Environ..

[14] Lin X., Jin Z.Y., Meng K., Zhang T.H., Zhao H.J. (2021). The development of flushable wipes. Tech. Text..

[15] Harter T., Steiner H., Bracic M., Kargl R., Hirn U. (2022). Deteriorating dispersibility of flushable wet wipes during storage: Role of fibre swelling and ionic shielding. J. Ind. Text..

[16] Xu Y.W., Ji Y.L., Ma J.H. (2023). Temperature-sensitive mussel-inspired citrate-based tissue adhesives with low-swelling. J. Adhes..

[17] Kim J.K., Park J.Y., Lee D.W., Ro D., Lee M.C., Han H.S. (2019). Temperature-sensitive anti-adhesive poloxamer hydrogel decreases fascial adhesion in total knee arthroplasty: A prospective randomized controlled study. J. Biomater. Appl..

[18] Mei Y., Che Q., Yang Q., Draper C., Yang H.P., Zhang S.H., Chen H.P. (2016). Torrefaction of different parts from a corn stalk and its effect on the characterization of products. Ind. Crop. Prod..

[19] Xu F., Zhong X.C., Sun R.C., Lu Q. (2006). Anatomy, ultrastructure and lignin distribution in cell wall of Caragana Korshinskii. Ind. Crop. Prod..

[20] (2009). Textiles—Test Methods for Nonwovens—Part 2: Determination of Thickness.

[21] (2009). Textiles—Test Methods for Nonwovens—Part 1: Determination of Mass per Unit Area.

[22] (2021). Test Method and Evaluation of Flushability of Disposable Hygienic Nonwoven Materials.

[23] (2010). Test Methods for Textiles Non-Woven Fabrics Part 6: Determination of Absorption.

[24] Wang D.M. (2007). Experimental discussion on the bleaching effect of sodium hypochlorite bleaching solution pH value and temperature on viscose filament. Artif. Fiber.

[25] Wu A.B. (2010). Bleaching Cotton Linter Pulp with Sodium Hypochlorite. Pap. Chem..

[26] Huang S., Zhou L., Li M.C., Wu Q.L., Zhou D.G. (2017). Cellulose Nanocrystals (CNCs) from Corn Stalk: Activation Energy Analysis. Materials.

[27] Durukan S., Karadagli F. (2020). Experimental data for physical characteristics, fiber compositions, and tensile properties of nonwoven wipes and toilet papers. Data Brief.

[28] Taylor L.D., Cerankowski L.D. (1975). Preparation of films exhibiting a balanced temperature dependence to permeation by aqueous solutions—A study of lower consolute behavior. J. Polym. Sci. Polym. Chem. Ed..

[29] Ruel-Gariépy E., Leroux J.C. (2004). In situ-forming hydrogels--review of temperature-sensitive systems. Eur. J. Pharm. Biopharm..

[30] Schild H.G. (1992). Poly(N-isopropylacrylamide): Experiment, theory and application. Prog. Polym. Sci..

[31] Park K. (1998). Controlled Drug Delivery: Challenges and strategies. J. Am. Chem. Soc..

[32] Fawaz F., Koffi A., Guyot M., Millet P. (2004). Comparative in vitro-in vivo study of two quinine rectal gel formulations. Int. J. Pharm..

[33] Michels L.R., Fachel F.N.S., Schuh R.S., Azambuja J.H., de Souza P.O., Gelsleichter N.E., Lenz G.S., Visioli F., Braganhol E., Teixeira H.F. (2023). Nasal administration of a temozolomide-loaded thermoresponsive nanoemulsion reduces tumor growth in a preclinical glioblastoma model. J. Control. Release.

[34] Wang C.J., Zhuo Y., Lu F.F., Chen B. (2013). Research progress on the gumming mechanism of temperature-sensitive gel materials. China Pharm..

[35] Wanka G., Hoffmann H., Ulbricht W. (1994). Phase diagrams and aggregation behavior of Poly(oxyethylene)-Poly(oxypropylene)-Poly(oxyethylene) triblock copolymers in aqueous solutions. Macromolecules.

[36] Jin H., Zha C., Gu L. (2007). Direct dissolution of cellulose in NaOH/thiourea/urea aqueous solution. Carbohydr. Res..

[37] Xu M. (2020). Application of corn stalk waste in the field of pulp and paper. East China Pulp. Pap. Ind..

