# Supplementary material for: Turning Waste into Treasure: The Full Technological Process and Product Performance Characterization of Flushable Wet Wipes Prepared from Corn Stalk
Source: Materials (Basel). 2023 Nov 16;16(22):7189. doi: 10.3390/ma16227189 (PMC10672815; doi:10.3390/ma16227189)
Supplement: Supplementary file 1 [file materials-16-07189-s001.zip › materials-2683905-supplementary.pdf]

Turning Waste into Treasure: The Full Technological Process and Product Performance Characterization of Flushable Wet Wipes Prepared from Corn Stalk

Lulu Liu †, Yeying Wang †, Ziying He, Yang Cai, Kai Meng, Ke-Qin Zhang and Huijing Zhao *

National Engineering Laboratory for Modern Silk, College of Textile and Clothing Engineering, Soochow University, No. 199 Ren'ai Road, Industrial Park, Suzhou 215123, China; liululu0819@163.com (L.L.); wangyeying0329@163.com (Y.W.); 20234215054@stu.suda.edu.cn (Z.H.); 2115408010@stu.suda.edu.cn (Y.C.); mk2009@suda.edu.cn (K.M.); kqzhang@suda.edu.cn (K.-Q.Z.)

* Correspondence: zhhj@suda.edu.cn

† These authors contributed equally to this work.

Alkali and bleaching treatment

As shown in Figure 1a, the yield of corn stalk pulp decreases with the increase of alkali concentration. The corn stalk components including ash, organic solvent extract and lignin can react with sodium hydroxide solution (NaOH) and be removed, therefore, the higher the alkali concentration, the greater the removal, and the lower the yield of corn stalk pulp[32]. As shown in Figure 1b, the yield of corn stalk pulp decreases with the increasing duration of alkali treatment. With increasing alkali treatment time, the sodium hydroxide solution (NaOH) reacted more fully with the corn stalk and the removal of lignin, so that the yield of corn stalk pulp decreased. As shown in Figure 1c, the yield of corn stalk pulp reduced as the alkali treatment temperature increased, but the decrease in yield is not obvious. It demonstrates that under the influence of alkali, high temperature also has a damaging effect on corn stalk, but the effect of temperature on it is less, the yield reduction is not obvious.

As shown in Figure 1d, the whiteness of corn stalk pulp showed a tendency to increase and then decrease with increasesing alkali treatment concentration. That is because higher alkali concentrations more thoroughly corn stalk is treated which conducive to bleaching. However, in a heated environment where the alkali concentration is too high, the decomposition of xylanan hemicellulose will be easier, and then xylose monomer is produced and oxidized, resulting in carbonyl chromophore and carbonyl color assist[33] , which will cause brightness reversion problems

in the corn stalk pulp. Figure 1-e shows that as the alkali treatment time increases, the whiteness of corn stalk pulp first increases and then decreases, the optimal alkali treatment time of 50 minutes. The longer the alkali treatment time, more thoroughly the lye reacts with corn stalks, the better the effect of bleaching, but the time is too long will cause serious fibre destruction, and the pulp begins to return to the yellow. The whiteness reached the highest value when the alkali treatment temperature was 90℃， as shown in Figure1-f. Because the higher the alkali treatment temperature, the faster the chemical reaction speed, the higher the component removal rate, the higher the degree of softening of corn stalk fiber, and the more conducive to bleaching.

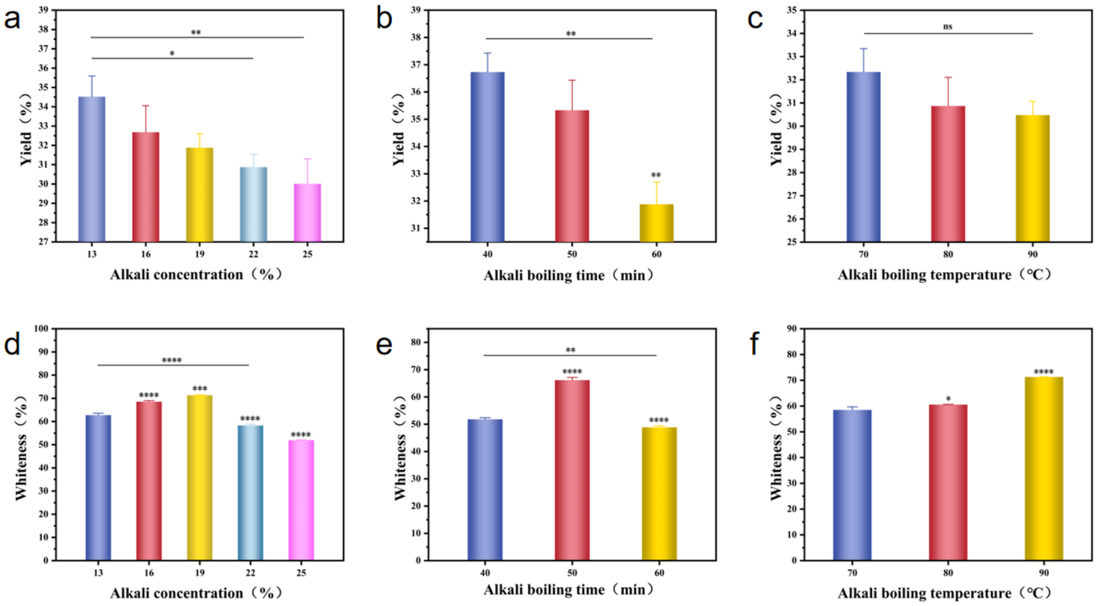

Figure S1. Effect of alkali treatment process on yield and whiteness of corn stalk pulp

References

32. Jin , H.; Zha, C.; Gu, L. Direct dissolution of cellulose in NaOH/thiourea/urea aqueous solution. Carbohydr. Res. 2007, 342, 851–858. https://doi.org/10.1016/j.carres.2006.12.023.

33. Xu, M. Application of corn stalk wa ste in the field of pulp and paper. East China Pulp. Pap. Ind. 2020, 50, 26–29.
